# Impact of soft-surface mobility exercises on functional movement screen (FMS) scores among football referees

**DOI:** 10.3389/fphys.2026.1757726

**Published:** 2026-02-25

**Authors:** Zülbiye Kaçay, Barış Baydemir, Laurentiu-Gabriel Talaghir, Gabriel Marian Manolache

**Affiliations:** 1 Faculty of Sport Sciences, Çanakkale Onsekiz Mart University, Çanakkale, Türkiye; 2 Faculty of Physical Education and Sport, Dunarea de Jos University of Galati, Galati, Romania

**Keywords:** football referees, functional movement screen, mobility, movement quality, neuromuscular control, soft-surface training, unstable surface training

## Abstract

**Introduction:**

Football referees perform repeated sprints, rapid changes of direction, and frequent deceleration–acceleration actions that place high demands on balance, mobility, and neuromuscular control. Limitations in functional movement patterns may negatively influence movement efficiency and increase mechanical stress during match officiating. This study aimed to examine the effects of a 12-week unstable/compliant surface–based mobility exercise program on functional movement quality, as assessed by the Functional Movement Screen (FMS), in young male amateur football referees.

**Methods:**

A total of 60 male amateur referees (experimental = 30; control = 30; age = 22.6 ± 1.3 years) participated in the study. Both groups continued their routine training, while the experimental group additionally performed mobility exercises on unstable and compliant surfaces (primarily BOSU-based drills) twice weekly for 12 weeks (45 ± 5 min/session). FMS tests were administered before and after the intervention. Data were analyzed using a 2 (group: experimental vs. control) × 2 (time: pre-test vs. post-test) mixed-effects ANOVA, with verification of normality through Shapiro–Wilk testing and visual inspection of residual distributions, and the group × time interaction was considered the primary indicator of intervention effectiveness.

**Results:**

The mixed ANOVA revealed significant group × time interaction effects for Total FMS score (p < 0.001) as well as for Deep Squat (p = 0.004), Hurdle Step (p < 0.001, partial η^2^ = 0.33), Active Straight Leg Raise (p = 0.043), Trunk Stability Push-up (p = 0.001), and Rotary Stability (p < 0.001). The control group showed minimal changes across all outcomes.

**Discussion:**

These findings indicate that unstable/compliant surface–based mobility training can improve movement quality indicators measured by the FMS. Incorporating such exercises into referee conditioning programs may contribute to more efficient movement patterns and enhanced dynamic stability, with potential implications for physical preparedness during match officiating.

## Introduction

Football is inherently a demanding sport. The fact that it is played on large fields, on different surfaces, and under various weather conditions requires individuals involved in the game to demonstrate high-level physical performance (Liu et al., 2025). One essential component of football is the referee, who plays a crucial role in managing the match and ensuring fair play. Football referees are required to sustain a high level of physical exertion comparable to that of players, regardless of environmental conditions (Liu et al., 2025; Baydemir et al., 2021). Unlike players, whose movements are influenced by ball location and playing position, referees must move across almost the entire field to maintain an optimal viewing angle and minimize decision-making errors.

During a 90-min match, referees typically cover approximately 10–12 km and perform repeated high-intensity actions such as accelerations, decelerations, directional changes, and backward running (Castagna et al., 2007; Weston et al., 2007). These continuous demands impose substantial mechanical load on the musculoskeletal system, and limitations in mobility, stability, or movement control may increase the likelihood of injury (Mallo et al., 2009). Therefore, improving movement quality and functional efficiency is highly relevant for referee performance and physical preparedness during match officiating. Movement quality reflects the efficiency and coordination with which fundamental movement patterns are executed and is closely linked to key motor abilities such as speed, agility, balance, and power. Previous research in team-sport athletes has demonstrated meaningful associations between functional movement quality and physical performance characteristics, indicating that higher-quality movement patterns may support more effective and economical execution of sport-specific tasks. These relationships have been demonstrated in various athletic populations, where FMS performance has been associated with speed, agility, jump performance, and selected anthropometric characteristics (Koźlenia et al., 2020; Alexe et al., 2024; Nicolozakes et al., 2018). Furthermore, functional movement quality has been linked to broader physical and health-related markers, suggesting that FMS performance may reflect multidimensional aspects of physical preparedness (Farrell et al., 2021; Cornell et al., 2017). In this context, assessing movement quality provides insight not only into potential injury risk factors but also into the functional foundation underpinning athletic performance.

Functional movement assessments are used to identify deficiencies and asymmetries in fundamental movement mechanics and to evaluate overall movement quality (Cook et al., 2014). In this context, the Functional Movement Screen (FMS) is a widely used tool for examining movement quality, highlighting limitations in mobility and stability, and identifying potential deficits in motor control (Cook et al., 2014). Previous research has shown that lower FMS scores may be associated with an increased risk of injury in athletic populations (Kiesel et al., 2007; Bonazza et al., 2017). Although this relationship has been examined primarily in players, evidence focusing on functional movement quality and FMS performance in football referees remains limited. Considering the high physical demands placed on referees during matches, assessing their movement quality and implementing targeted training programs may be particularly important (O'Connor et al., 2011).

Mobility and stability constitute the core components of functional movement ability. Mobility refers to muscle flexibility and joint range of motion, whereas stability involves postural control and balance. Adequate mobility may enhance movement efficiency during dynamic actions such as rapid direction changes, accelerations, and abrupt stops, while insufficient mobility or stability may increase the risk of injury (Hrysomallis, 2013). These components are fundamental targets of functional training interventions aimed at improving movement quality and neuromuscular control. Traditional mobility exercises are commonly performed on stable or rigid surfaces. However, training on soft or compliant surfaces may provide additional proprioceptive challenges and contribute to improvements in postural stability and neuromuscular control (Paillard, 2012). Such training environments may stimulate sensorimotor adaptations that support movement coordination and functional performance, potentially leading to improvements in functional movement patterns and FMS outcomes (Winter et al., 2022). Given the documented associations between functional movement quality and motor performance parameters in team-sport athletes, interventions targeting mobility and stability under increased neuromuscular demand may have practical relevance beyond isolated movement screening scores (Koźlenia et al., 2020; Alexe et al., 2024).

Despite these potential benefits, the effects of structured mobility exercises performed on soft surfaces on FMS scores have not been sufficiently investigated, particularly in football referees. Moreover, it remains unclear to what extent mobility training performed under unstable or compliant conditions can enhance movement quality as reflected by standardized functional assessments rather than isolated performance metrics. Given the high match demands experienced by referees, examining the impact of soft-surface mobility training on movement quality may provide a scientific basis for designing more effective conditioning strategies that support functional efficiency and neuromuscular preparedness. Based on the theoretical framework linking mobility, stability, and neuromuscular control to functional movement quality, it was hypothesized that:

1. A 12-week unstable/compliant surface–based mobility exercise program would result in significantly greater improvements in total FMS score in the experimental group compared with the control group; and
2. Significant group × time interaction effects would be observed for key FMS components associated with lower-extremity mobility, balance, and core stability (e.g., Deep Squat, Hurdle Step, Trunk Stability Push-up, and Rotary Stability).

## Methods

### Research group

The study included a total of 60 male football referees (30 experimental, 30 control) officiating in amateur leagues. The research was conducted in accordance with the principles of the Declaration of Helsinki. Ethical approval was obtained from the Non-Interventional Clinical Research Ethics Committee of Çanakkale Onsekiz Mart University (Approval No: 2025-49), and all participants provided written informed consent prior to participation. Participants were eligible for inclusion if they were actively officiating as football referees in amateur leagues, had at least 1 year of refereeing experience, and were free from musculoskeletal injury at the time of data collection. Referees were excluded if they reported any acute injury, neurological disorder, or medical condition that could affect movement performance or participation in physical training. Participants were allocated to the experimental and control groups using a non-randomized, convenience-based allocation procedure determined by training availability and scheduling constraints. Randomization was not feasible due to league-based training logistics; however, baseline characteristics were comparable between groups (Table 1). The control group continued their routine training and did not participate in the soft-surface mobility program. A total of 60 referees were initially recruited, and all participants completed the study protocol. No dropouts or missing data were recorded during the 12-week intervention period.

**TABLE 1:** Descriptive characteristics of the football referees at baseline (Mean ± SD).

Variable	Control (n = 30) mean ± SD	Experimental (n = 30) mean ± SD
Age (years)	22.60 ± 1.35	22.50 ± 1.31
Height (cm)	181.17 ± 4.68	181.47 ± 4.20
Body weight (kg)	77.37 ± 4.53	74.40 ± 6.21
BMI (kg/m^2^)	23.51 ± 1.38	22.42 ± 1.90

Values are presented as mean ± standard deviation (SD). BMI, body mass index (kg/m^2^). Control group: n = 30; Experimental group: n = 30.

### Implementation of soft-surface mobility exercises

During the 12-week intervention period, the football referees in the experimental group performed soft-surface mobility exercises twice per week for approximately 45 ± 5 min per session. The program aimed to improve joint range of motion, muscular flexibility, proprioception, balance, and dynamic stability through exercises performed on unstable and compliant surfaces.

The training program consisted of two weekly sessions (Session A and Session B), including a standardized warm-up (5–10 min), a main exercise section (∼30 min), and a cool-down phase (5–10 min). The exercises included progressive mobility and stability drills using BOSU-based movements (e.g., BOSU squat, BOSU lunge, BOSU glute bridge, BOSU plank, BOSU bird-dog), with gradual progression in training volume and duration throughout the 12 weeks. The full intervention protocol is provided in Supplementary Table S1.

In brief, during the first 3 weeks, the sessions focused on fundamental mobility drills, joint range-of-motion development, and core activation. From weeks four to six, balance, stabilization, and proprioception-oriented exercises were introduced. Between the seventh and ninth weeks, the program emphasized mobility exercises targeting the hip, knee, and ankle joints. In the final stage (weeks 10–12), dynamic movements combining mobility and agility were incorporated (Nepocatych et al., 2018; Zech et al., 2010).

Exercise sessions were supervised by the research team, and attendance was recorded for each session using a standardized participation log. All participants completed the 12-week protocol, resulting in 100% adherence and no attrition. Intervention fidelity was ensured through direct supervision during all sessions. Correct exercise execution was monitored visually by the supervising researchers, and verbal feedback was provided when necessary to maintain proper technique. Session attendance was recorded using signed participation logs, and all referees attended all scheduled training sessions, resulting in full adherence to the intervention protocol.

The term “soft-surface” was used as a general category referring to compliant and unstable training conditions (e.g., BOSU devices, grass, sand, tatami). Surface-specific effects were not analyzed separately, as the aim of the intervention was to induce generalized proprioceptive and neuromuscular challenges rather than isolate individual surface characteristics.

### Data collection procedures

#### Anthropometric measurements

The height and body weight of the referees were measured, and Body Mass Index (BMI) was calculated. A Dikomsan BW-200 digital scale and stadiometer were used for all anthropometric assessments. Measurements were taken in the morning under standardized conditions. BMI was computed using the following formula:

$$\text{BMI}=\text{body mass }\left(\text{kg}\right)\;/\;{\text{height}}^{2}\;\left(m^{2}\right).$$

(Benazeera, 2014).

#### Functional movement screening (FMS)

The Functional Movement Screen (FMS) was used to assess functional movement quality. The FMS consists of seven movement patterns, each scored on a scale from 0 to 3, and the total FMS score ranges from 0 to 21, representing the sum of all individual scores (Cook et al., 2006). Each movement was scored based on established normative criteria (Bardenett et al., 2015; Baydemir and Dilican, 2025).

FMS scoring was performed by two trained raters (academics working in the field of Sport Sciences) who had received formal training in FMS assessment and scoring procedures. All tests were conducted using direct observation. Each rater scored the participants independently and blinded to the other rater’s scores, and the mean of the two raters’ scores was used for statistical analysis. The same two raters performed both the pre-test and post-test assessments to ensure scoring consistency across time.

Although both raters were formally trained and adhered to standardized FMS scoring guidelines, formal inter-rater reliability statistics (e.g., intraclass correlation coefficients) were not calculated. Individual rater-level scores were not retained following computation of the mean score used for statistical analyses; therefore, inter-rater ICC values could not be calculated retrospectively. This approach was chosen to maintain scoring consistency across testing sessions by using identical raters at both time points; however, the absence of empirical reliability indices is acknowledged as a methodological limitation.

### Statistical analysis

*A priori* power analysis was conducted using G*Power (α = 0.05, power = 0.80, medium effect size f = 0.25) for a mixed ANOVA design (group × time), indicating that a minimum sample size of 54 participants was required. Prior to the main analyses, missing-value and outlier diagnostics were performed; no missing or extreme values were detected, and descriptive statistics were subsequently computed. Normality of the outcome variables was assessed using the Shapiro–Wilk test and visual inspection methods (e.g., histograms and Q–Q plots) (González-Estrada and Cosmes, 2019). Model residuals were visually inspected to confirm approximate normality. Although individual FMS component scores are ordinal in nature (0–3), they were treated as quasi-continuous variables for statistical analysis, consistent with previous FMS-based research employing parametric methods in similar designs (Kiesel et al., 2007; Frost et al., 2015; Bonazza et al., 2017; Uysal and Baydemir, 2026). The main analysis was conducted using a 2 (group: experimental vs. control) × 2 (time: pre-test vs. post-test) mixed ANOVA for each FMS outcome, with the group × time interaction considered the primary indicator of the intervention effect. Effect sizes were reported using partial eta squared (partial η^2^). Statistical significance was set at p < 0.05.

## Results

**FIGURE 1:**
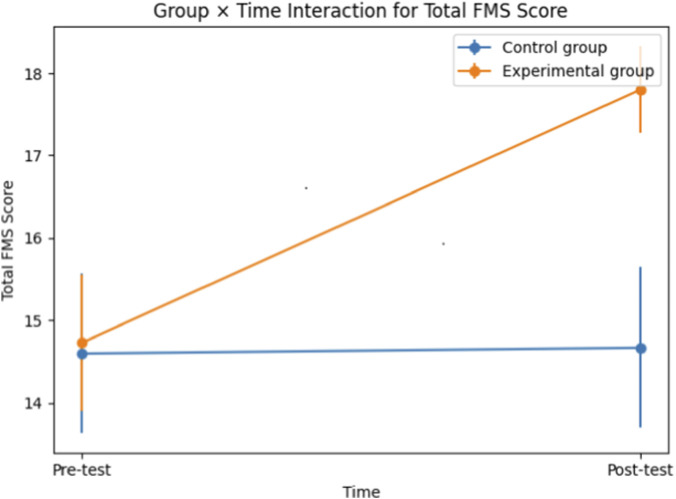
Group × time interaction for total Functional Movement Screen (FMS) score in the experimental and control groups. Values are presented as means with 95% confidence intervals.

Participant characteristics at baseline are presented in Table 1. The experimental and control groups showed comparable demographic and anthropometric profiles. Pre- and post-intervention FMS component scores and total FMS scores are summarized in Table 2. The control group demonstrated minimal changes from pre-test to post-test across all FMS outcomes, whereas the experimental group showed noticeable improvements in several FMS components and in total FMS score. The 2 (group: experimental vs. control) × 2 (time: pre-test vs. post-test) mixed ANOVA revealed statistically significant group × time interaction effects for Total FMS score (p < 0.001) as well as for Deep Squat (p = 0.004, partial η^2^ = 0.14), Hurdle Step (p < 0.001, partial η^2^ = 0.49), Active Straight Leg Raise (p = 0.043, partial η^2^ = 0.07), Trunk Stability Push-up (p = 0.001, partial η^2^ = 0.17), and Rotary Stability (p < 0.001, partial η^2^ = 0.42) (Table 3). These interaction effects indicate that the experimental group demonstrated significantly greater improvements over time compared with the control group, with effect sizes ranging from moderate to large depending on the FMS component. The group × time interaction for total FMS score is illustrated in Figure 1. In contrast, the group × time interaction effect was not statistically significant for In-line Lunge (p = 0.206, partial η^2^ = 0.03) and Shoulder Mobility (p = 0.689, partial η^2^ < 0.01) (Table 3), indicating comparable pre-to post-test changes between groups for these movement patterns.

**TABLE 2:** Pre–post intervention FMS component and total scores in the experimental and control groups (Mean ± SD).

Outcome	Control (Pre) (n = 30)	Control (Post) (n = 30)	Experimental (Pre) (n = 30)	Experimental (Post) (n = 30)
Deep squat (score)	2.10 ± 0.84	2.03 ± 0.85	2.07 ± 0.78	2.53 ± 0.51
Hurdle step (score)	1.33 ± 0.61	1.33 ± 0.61	1.37 ± 0.61	2.30 ± 0.47
In-line lunge (score)	1.97 ± 0.49	1.97 ± 0.49	2.00 ± 0.45	2.13 ± 0.35
Shoulder mobility (score)	2.40 ± 0.72	2.37 ± 0.72	2.47 ± 0.68	2.50 ± 0.51
Active straight leg raise (score)	1.80 ± 0.55	1.80 ± 0.55	1.83 ± 0.53	2.13 ± 0.35
Trunk stability push-up (score)	1.73 ± 0.94	1.80 ± 0.96	1.80 ± 0.96	2.53 ± 0.51
Rotary stability (score)	1.27 ± 0.64	1.30 ± 0.65	1.23 ± 0.63	2.17 ± 0.38
Total FMS (score)	14.60 ± 2.71	14.67 ± 2.72	14.73 ± 2.30	17.80 ± 1.47

Values are presented as mean ± standard deviation (SD). FMS, Functional Movement Screen. All outcomes are expressed as FMS, scores (0–3 for each movement pattern). Control group: n = 30; Experimental group: n = 30. Higher scores indicate better movement quality.

**TABLE 3:** Group × time interaction effects (intervention effect) for FMS outcomes (Mixed design: Experimental vs. Control × Pre vs. Post).

Outcome	F(1,58)	p-value	Partial η^2^
Deep squat (score)	9.05	0.004	0.14
Hurdle step (score)	54.65	<0.001	0.49
In-line lunge (score)	1.63	0.206	0.03
Shoulder mobility (score)	0.16	0.689	0.00
Active straight leg raise (score)	4.28	0.043	0.07
Trunk stability push-up (score)	11.46	0.001	0.17
Rotary stability (score)	41.86	<0.001	0.42
Total FMS (score)	28.60	<0.001	0.33

Group = experimental vs. control (between-subject factor). Time = pre-test vs. post-test (within-subject factor). F = F-statistics. Partial η^2^ = effect size. A significant group × time interaction indicates that the experimental group improved more than the control group over time.

Figure 1 illustrates the group × time interaction for total FMS score, showing a marked increase in the experimental group from pre-to post-test, while the control group remained relatively stable.

## Discussion

The present study investigated the effects of a 12-week unstable/compliant surface–based mobility exercise program on Functional Movement Screen (FMS) performance in male football referees. The main finding of this study was that the experimental group demonstrated significantly greater improvements than the control group in total FMS score, suggesting that this type of mobility training is associated with improvements in movement quality indicators measured by the FMS.

In addition to improvements in total FMS score, the mixed ANOVA results indicated significant group × time interaction effects for key movement components including Deep Squat, Hurdle Step, Active Straight Leg Raise, Trunk Stability Push-up, and Rotary Stability. These outcomes reflect improvements in lower-extremity mobility, dynamic balance, core stability, and rotational control, which are essential physical qualities for football referees due to the frequent accelerations, decelerations, directional changes, and multi-planar movements required during match officiating. Referees perform under repeated high-intensity demands, and efficient movement patterns may support more economical movement execution during match activities.

From a physiological and neuromuscular perspective, the observed improvements may be explained by the specific demands imposed by training on unstable and compliant surfaces. Exercises performed under unstable conditions increase postural challenge and modify afferent sensory input from mechanoreceptors located in muscles, tendons, and joint structures. This enhanced sensory feedback promotes greater sensorimotor integration within the central nervous system, facilitating improved coordination between agonist, antagonist, and stabilizing muscle groups (Paillard, 2012; Zech et al., 2010; Winter et al., 2022).

Furthermore, soft-surface training has been shown to increase activation of deep stabilizing musculature and to enhance trunk control through both feedforward and feedback motor control mechanisms. Improved feedforward activation may allow referees to better anticipate perturbations during rapid changes of direction, whereas enhanced feedback control contributes to effective postural corrections during unexpected balance challenges. These neuromuscular adaptations may explain the substantial improvements observed in trunk stability and rotary stability FMS components, which are particularly sensitive to deficits in core control and intermuscular coordination.

Improvements in lower-extremity mobility-related components such as the Deep Squat and Active Straight Leg Raise may also be attributed to enhanced joint range of motion and reduced neuromuscular inhibition following repeated exposure to controlled instability. Training on compliant surfaces may promote more efficient regulation of joint stiffness and improved intermuscular coordination, allowing referees to execute complex movement patterns with greater symmetry and control.

The findings of this study are in line with previous literature suggesting that balance- and mobility-oriented training programs can positively influence functional movement performance (Zech et al., 2010; Paillard, 2012; Winter et al., 2022). The FMS has been widely used to assess movement quality and identify potential limitations in fundamental movement patterns. Given that football referees operate in environments requiring high levels of multi-joint coordination, improving movement quality through targeted mobility and stability training may provide practical benefits for performance preparation. Therefore, incorporating soft-surface mobility exercises into referee conditioning routines may be considered a feasible and sport-specific approach to support functional movement capacity in this population.

## Conclusion

In conclusion, a 12-week unstable/compliant surface–based mobility exercise program performed twice weekly significantly improved total FMS score and several key movement components compared with the control condition.

These findings suggest that this form of mobility training is associated with improvements in functional movement quality, dynamic balance, and neuromuscular control in football referees.

Incorporating such training into referee conditioning programs may support movement efficiency and physical preparedness for match demands; however, direct effects on performance or injury outcomes were not assessed in the present study.

## Limitations

This study has several limitations. First, the sample consisted of male amateur football referees, which may limit the generalizability of the findings to female referees or elite-level officials. Second, although FMS scoring was conducted independently by two trained raters and the mean score was used for analysis, formal inter-rater reliability statistics (e.g., intraclass correlation coefficients) were not calculated. While rater training and standardized scoring procedures help reduce measurement error, the absence of empirical reliability estimates limits confidence in the objectivity and reproducibility of the FMS scores. Furthermore, because individual rater-level data were not archived, inter-rater agreement statistics could not be computed *post hoc*, and future studies should retain individual scoring sheets to allow formal ICC calculation. Third, FMS component scores are ordinal measures; although they were treated as quasi-continuous variables in accordance with common practice in the FMS literature, future studies may benefit from complementary biomechanical or kinematic assessments to strengthen interpretation of movement-quality changes. Finally, the absence of long-term follow-up precludes conclusions regarding the retention of observed improvements over time.

## Practical implications and future directions

These findings suggest that incorporating soft-surface mobility exercises into referee conditioning programs may enhance movement quality and functional stability, with potential implications for movement efficiency during match officiating. Future studies should examine long-term retention of these adaptations, include female and professional referees, and compare different training surfaces and intensity progressions to optimize functional movement outcomes. Additionally, future research incorporating direct performance metrics and injury surveillance would help clarify the practical significance of improvements in functional movement quality.

## Data Availability

The raw data supporting the conclusions of this article will be made available by the authors, without undue reservation.

## References

[B1] Alexe D. I. Čaušević D. Čović N. Rani B. Tohănean D. I. Abazović E. (2024). The relationship between functional movement quality and speed, agility, and jump performance in elite female youth football players. Sports 12 (8), 214. 10.3390/sports12080214 39195590 PMC11359347

[B2] Bardenett S. M. Micca J. J. DeNoyelles J. T. Miller S. D. Jenk D. T. Brooks G. S. (2015). Functional movement screen normative values and validity in high school athletes: can the fms^TM^ be used as A predictor of injury? Int. Journal Sports Physical Therapy 10 (3), 303–308. 26075145 PMC4458917

[B3] Baydemir B. Dilican T. (2025). Ankle mobility and functional movement abilities in football referees: a correlational study. J. Phys. Educ. Sports Stud. 17 (2), 130–143. 10.55929/besad.1663600

[B4] Baydemir B. Yurdakul H. O. Aksoy S. (2021). The effect of different training strategies applied to football referees on VO2max and running performance. Pak. J. Med. and Health Sci. 15 (10), 2933–2937. 10.53350/pjmhs2115102933

[B5] Benazeera U. J. (2014). Association between eating habits and body mass index (BMI) of adolescents. Int. J. Med. Sci. Public Health 3 (8), 940–943.

[B6] Bonazza N. A. Smuin D. Onks C. A. Silvis M. L. Dhawan A. (2017). Reliability, validity, and injury predictive value of the functional movement screen: a systematic review and meta-analysis. The *American Journal of Sports Medicine* . Am. J. Sports Med. 45 (3), 725–732. 10.1177/0363546516641937 27159297

[B7] Castagna C. Abt G. D’Ottavio S. (2007). Physiological aspects of soccer refereeing performance and training. Sports Med. 37 (7), 625–646. 10.2165/00007256-200737070-00006 17595157

[B8] Cook G. Burton L. Hoogenboom B. (2006). Pre-participation screening: the use of fundamental movements as an assessment of function—Part 1. North Am. J. Sports Phys. Ther. 1 (2), 62–70. 21522216 PMC2953313

[B9] Cook G. Burton L. Hoogenboom B. J. Voight M. (2014). Functional movement screening: the use of fundamental movements as an assessment of function—Part 1. Int. J. Sports Phys. Ther. 9 (3), 396–409. 24944860 PMC4060319

[B10] Cornell D. J. Gnacinski S. L. Zamzow A. Mims J. Ebersole K. T. (2017). Measures of health, fitness, and functional movement among firefighter recruits. Int. J. Occup. Saf. Ergonomics 23 (2), 198–204. 10.1080/10803548.2016.1187001 27191666

[B11] Farrell S. W. Pavlovic A. Barlow C. E. Leonard D. DeFina J. R. Willis B. L. (2021). Functional movement screening performance and Association with key health markers in older adults. J. Strength Cond. Res. 35 (11), 3021–3027. 10.1519/JSC.0000000000003273 31895281

[B12] Frost D. M. Beach T. A. Callaghan J. P. McGill S. M. (2015). FMS scores change with Performers Knowledge of the Grading Criteria-Are General Whole-Body Movement Screens Capturing Dysfunction. J. Strength Cond. Res. 29 (11), 3037–3044. 10.1097/JSC.0000000000000211 26502271

[B13] González-Estrada E. Cosmes W. (2019). Shapiro–Wilk test for skew normal distributions based on data transformations. J. Stat. Comput. Simul. 89 (17), 3258–3272. 10.1080/00949655.2019.1658763

[B14] Hrysomallis C. (2013). Injury incidence, risk factors and prevention in Australian rules football. Sports Med. 43 (5), 339–354. 10.1007/s40279-013-0034-0 23529288

[B15] Kiesel K. Plisky P. J. Voight M. L. (2007). Can serious injury in professional football be predicted by a preseason functional movement screen? North Am. Journal Sports Physical Therapy 2 (3), 147–158. 21522210 PMC2953296

[B16] Koźlenia D. Trojanowska I. Domaradzki J. Czermak P. (2020). Association between speed and agility abilities with movement patterns quality in team sports players. Med. Dello Sport 73 (2), 176–186. 10.23736/S0025-7826.20.03662-5

[B17] Liu X. Zuo Y. Aslam H. S. Kim S. Fu M. (2025). Exploring factors influencing number of fouls in soccer. Front. Psychol. 16, 1510928. 10.3389/fpsyg.2025.1510928 40463301 PMC12131515

[B18] Mallo J. Navarro E. Aranda J. M. G. Helsen W. F. (2009). Activity profile of top-class association football referees in relation to fitness-test performance and match standard. J. Sports Sci. 27 (1), 9–17. 10.1080/02640410802298227 18979338

[B19] Nepocatych S. Ketcham C. J. Vallabhajosula S. Balilionis G. (2018). The effects of unstable surface balance training on postural sway, stability, functional ability and flexibility in women. J. Sports Medicine Physical Fitness 58 (1-2), 27–34. 10.23736/S0022-4707.16.06797-9 27991482

[B20] Nicolozakes C. P. Schneider D. K. Roewer B. D. Borchers J. R. Hewett T. E. (2018). Influence of body composition on functional movement screen^TM^ scores in college football players. J. Sport Rehabilitation 27 (5), 431–437. 10.1123/jsr.2015-0080 28714791

[B21] O'Connor F. G. Deuster P. A. Davis J. Pappas C. G. Knapik J. J. (2011). Functional movement screening: predicting injuries in officer candidates. Med. Science Sports Exercise 43 (12), 2224–2230. 10.1249/MSS.0b013e318223522d 21606876

[B22] Paillard T. (2012). Effects of general and local fatigue on postural control: a review. Neurosci. and Biobehav. Rev. 36 (1), 162–176. 10.1016/j.neubiorev.2011.05.009 21645543

[B23] Uysal G. E. Baydemir B. (2026). Functional movement screen and asymmetries in female volleyball players across playing positions. Sci. Rep. 16, 4979. 10.1038/s41598-026-35725-w 41520037 PMC12876947

[B24] Weston M. Castagna C. Impellizzeri F. M. Rampinini E. Abt G. (2007). Analysis of physical match performance in English Premier League soccer referees with particular reference to first half and player work rates. J. Sci. Med. Sport 10 (6), 390–397. 10.1016/j.jsams.2006.09.001 17126077

[B25] Winter L. Huang Q. Sertic J. V. Konczak J. (2022). The effectiveness of proprioceptive training for improving motor performance and motor dysfunction: a systematic review. Front. Rehabilitation Sciences 3, 830166. 10.3389/fresc.2022.830166 36188962 PMC9397687

[B26] Zech A. Hübscher M. Vogt L. Banzer W. Hänsel F. Pfeifer K. (2010). Balance training for neuromuscular control and performance enhancement: a systematic review. J. Athl. Train. 45 (4), 392–403. 10.4085/1062-6050-45.4.392 20617915 PMC2902034

