# Supplementary material for: Impact of soft-surface mobility exercises on functional movement screen (FMS) scores among football referees
Source: Front Physiol. 2026 Feb 25;17:1757726. doi: 10.3389/fphys.2026.1757726 (PMC12975583; doi:10.3389/fphys.2026.1757726)
Supplement: Supplementary file 1 [file Table1.docx]

**Supplementary Table 1. Soft-surface mobility exercise program (12-week protocol)**

Supplementary Table 1. Detailed 12-week soft-surface mobility exercise program performed by the experimental group (2 sessions/week).

**Note:** Each session included warm-up (5–10 min), main exercises (~30 min), and cool-down (5–10 min). Warm-up consisted of dynamic stretching (e.g., leg swings, shoulder circles) and cool-down consisted of static stretching.

**Weeks 1–2**

**Session A (Day 1)**

- BOSU Squat 3 sets × 10–12 reps
- BOSU Lunge 3 sets × 10 reps each leg
- BOSU Glute Bridge 3 sets × 12 reps
- BOSU Push-up 3 sets × 10 reps

**Session B (Day 2)**

- BOSU Plank 3 sets × 30 s
- BOSU Side Lunge3 sets × 12 reps each leg
- BOSU Glute Bridge 3 sets × 12–15 reps
- BOSU Bird Dog 3 sets × 12 reps each leg

**Weeks 3–4**

**Session A**

- BOSU Squat 3 sets × 12–15 reps
- BOSU Lunge 3 sets × 12 reps each leg
- BOSU Glute Bridge 3 sets × 15 reps
- BOSU Push-up 3 sets × 12 reps

**Session B**

- BOSU Plank 3 sets × 45 s
- BOSU Side Lunge 3 sets × 15 reps each leg
- BOSU Glute Bridge 3 sets × 15–20 reps
- BOSU Bird Dog 3 sets × 15 reps each leg

**Weeks 5–6**

**Session A**

- BOSU Squat 3 sets × 15 reps
- BOSU Lunge 3 sets × 12–15 reps each leg
- BOSU Glute Bridge 3 sets × 15–20 reps
- BOSU Push-up 3 sets × 15 reps

**Session B**

- BOSU Plank 3 sets × 45 s
- BOSU Side Lunge 3 sets × 15 reps each leg
- BOSU Glute Bridge 3 sets × 20 reps
- BOSU Bird Dog 3 sets × 15 reps each leg

**Weeks 7–8**

**Session A**

- BOSU Squat 3 sets × 20–25 reps
- BOSU Lunge 3 sets × 15–20 reps each leg
- BOSU Glute Bridge 3 sets × 20–25 reps
- BOSU Push-up 3 sets × 20 reps

**Session B**

- BOSU Plank 3 sets × 60 s
- BOSU Side Lunge 3 sets × 20 reps each leg
- BOSU Glute Bridge 3 sets × 25 reps
- BOSU Bird Dog 3 sets × 20 reps each leg

**Weeks 9–10**

**Session A**

- BOSU Squat 3 sets × 20–25 reps
- BOSU Lunge 3 sets × 15–20 reps each leg
- BOSU Glute Bridge 3 sets × 20–25 reps
- BOSU Push-up 3 sets × 20 reps

**Session B**

- BOSU Plank 3 sets × 60 s
- BOSU Side Lunge 3 sets × 20 reps each leg
- BOSU Glute Bridge 3 sets × 25 reps
- BOSU Bird Dog 3 sets × 20 reps each leg

**Weeks 11–12**

**Session A**

- BOSU Squat 3 sets × 15 reps
- BOSU Lunge 3 sets × 12–15 reps each leg
- BOSU Glute Bridge 3 sets × 15–20 reps
- BOSU Push-up 3 sets × 15 reps

**Session B**

- BOSU Plank 3 sets × 45 s
- BOSU Side Lunge 3 sets × 15 reps each leg
- BOSU Glute Bridge 3 sets × 20 reps
- BOSU Bird Dog 3 sets × 15 reps each leg
